# Cardiovascular magnetic resonance imaging characteristics of a cardiac myeloid sarcoma with superimposed thrombus

**DOI:** 10.1093/ehjci/jeag097

**Published:** 2026-04-15

**Authors:** Michiel J Bom, Lisanne Lutter, Mette D Hazenberg, Brian V Balgobind, Lourens F H J Robbers, Ramon B van Loon

**Affiliations:** Department of Cardiology, Amsterdam UMC, Vrije Universiteit Amsterdam, De Boelelaan 1117, 1081 HV Amsterdam, The Netherlands; Department of Pathology, Amsterdam UMC, Vrije Universiteit Amsterdam, Amsterdam, The Netherlands; Department of Hematology, Cancer Center Amsterdam, Amsterdam UMC, University of Amsterdam, Amsterdam, the Netherlands; Department of Radiation Oncology, Cancer Center Amsterdam, Amsterdam UMC, University of Amsterdam, Amsterdam, The Netherlands; Department of Cardiology, Amsterdam UMC, Vrije Universiteit Amsterdam, De Boelelaan 1117, 1081 HV Amsterdam, The Netherlands; Department of Cardiology, Amsterdam UMC, Vrije Universiteit Amsterdam, De Boelelaan 1117, 1081 HV Amsterdam, The Netherlands

**Figure:**
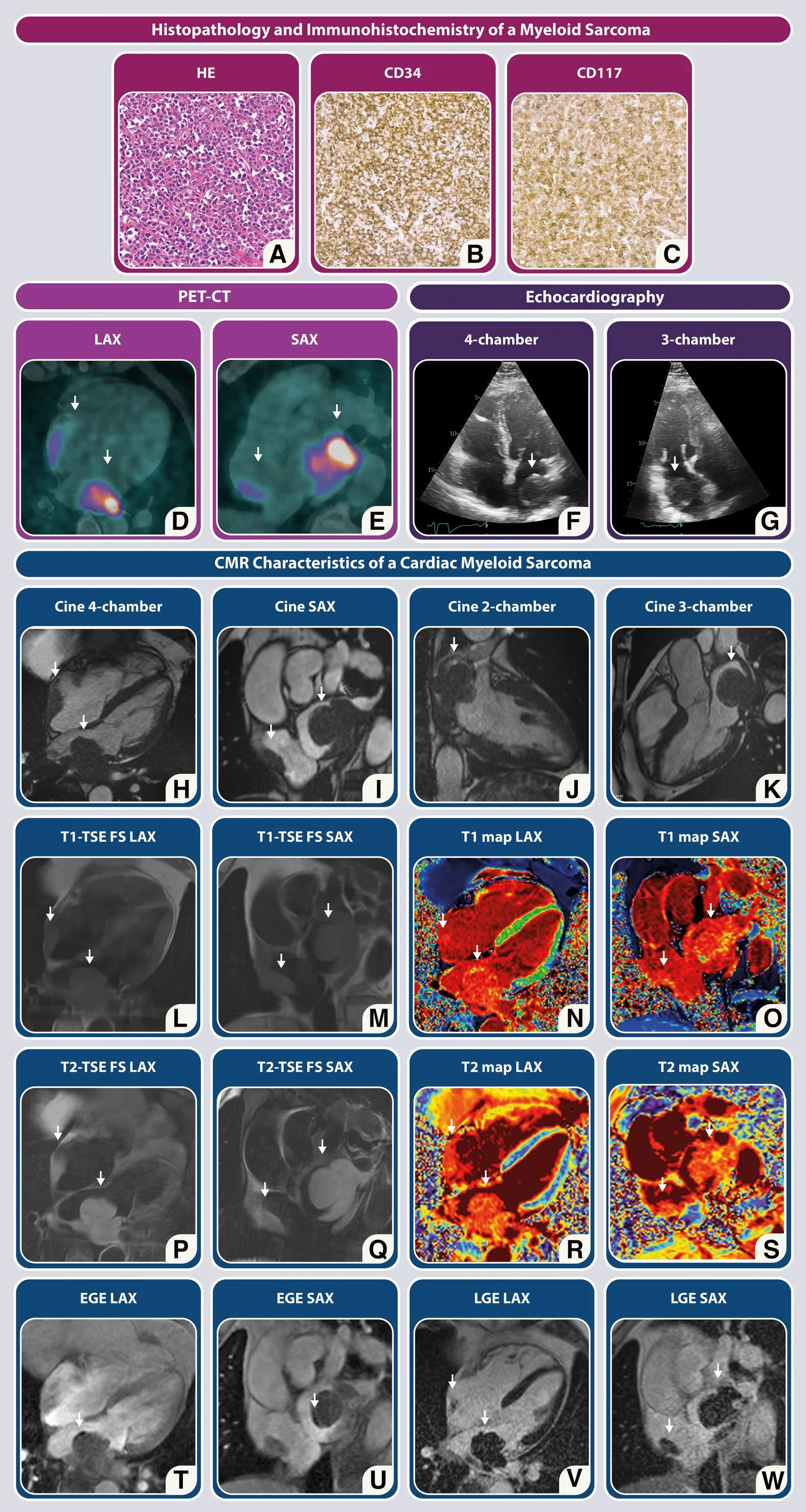


A patient with prior myelofibrosis for which he received allogenic hematopoetic progenitor cell transplantation 7 years earlier developed a distal left femur tumour. Histopathology and immunohistochemistry of the operatively resected mass was consistent with myeloid sarcoma (MS), an extramedullary tumour consisting of myeloid blasts expressing CD34 and CD117 (Panels *A*–*C*). Additional positron emission tomography-computed tomography (PET-CT) revealed increased tracer uptake in both atria suggestive of a cardiac MS (Panels *D* and *E*). The patient had no cardiac symptoms. Echocardiography demonstrated an immobile left atrial mass (Panels *F* and *G*). Cardiac magnetic resonance (CMR) was performed for tissue characterization. Cine-images showed a multilobular left atrial mass (measuring 46 × 36 mm) with a thickened atrial wall (Panels *H*–*K*) and a separate right atrial mass (measuring 11 × 43 mm) along the lateral wall with pericardial thickening. Both lesions were isointense compared with the myocardium on T1-weighed images (Panels *L* and *M*) and had high T1 values on parametric mapping (1344–1374 ms, Panels *N* and *O*) consistent with increase in free water or fibrosis typically seen in malignant masses. Furthermore, both lesions were hyperintense on T2-weighed images (Panels *P* and *Q*) with high T2 values on parametric mapping (87–88 ms, Panels *R* and *S*) consistent with oedema. Early gadolinium enhancement (EGE) images showed hypointense areas on the left atrial mass suggestive of superimposed thrombus (Panels *T* and *U*). Lastly, late gadolinium enhancement (LGE) demonstrated heterogeneous LGE reflecting interstitial fibrosis or myocyte necrosis (Panels *V* and *W*). Since MS are sensitive to radiotherapy the patient received a single fraction of 8 Gy on both masses.

Concluding, in patients with suspected cardiac MS, CMR offers important insights in tissue characterization, extent of disease and the presence of thrombi.

Supplementary data are available at *European Heart Journal - Cardiovascular Imaging* online.

**Author contributions:** Michiel J Bom [Visualization, Writing—original draft (lead)], Lisanne Lutter [Visualization, Writing—original draft, Writing—review & editing (supporting)], Mette D Hazenberg [Writing—review & editing (supporting)], Brian V Balgobind [Writing—review & editing (supporting)], Lourens F.H.J. Robbers [Visualization, Writing—review & editing (supporting)], and Ramon B Van Loon [Supervision, Writing—review & editing (lead)]

**Funding:** None declared.

**Data availability:** No new data were generated or analysed in support of this article.

## Supplementary Material

jeag097_Supplementary_Data

